# The Photostability of Novel Boron Hydride Blue Emitters in Solution and Polystyrene Matrix

**DOI:** 10.3390/ma14030589

**Published:** 2021-01-27

**Authors:** Jakub Ševčík, Pavel Urbánek, Barbora Hanulíková, Tereza Čapková, Michal Urbánek, Jan Antoš, Michael G. S. Londesborough, Jonathan Bould, Bita Ghasemi, Lukáš Petřkovský, Ivo Kuřitka

**Affiliations:** 1 Centre of Polymer Systems, Tomas Bata University in Zlín, třída Tomáše Bati 5678, 760 01 Zlín, Czech Republic; j4sevcik@utb.cz (J.Š.); hanulikova@utb.cz (B.H.); capkova@utb.cz (T.Č.); murbanek@utb.cz (M.U.); antos@utb.cz (J.A.); ghasemi@utb.cz (B.G.); l_petrkovsky@utb.cz (L.P.); 2 Institute of Inorganic Chemistry, Academy of Sciences of the Czech Republic, 250 68 Husinec-Řež, Czech Republic; michaell@iic.cas.cz (M.G.S.L.); jbould@gmail.com (J.B.)

**Keywords:** boronhydride emitters, *anti*-B_18_H_22_, photostability, OLED

## Abstract

In recent work, the boron hydride *anti*-B_18_H_22_ was announced in the literature as a new laser dye, and, along with several of its derivatives, its solutions are capable of delivering blue luminescence with quantum yields of unity. However, as a dopant in solid polymer films, its luminescent efficiencies reduce dramatically. Clarification of underlying detrimental effects is crucial for any application and, thus, this contribution makes the initial steps in the use of these inorganic compounds in electrooptical devices based on organic polymer thin films. The photoluminescence behavior of the highly luminescent boron hydrides, *anti*-B_18_H_22_ and 3,3′,4,4′-Et_4_-*anti*-B_18_H_18_, were therefore investigated. The quantum yields of luminescence and photostabilities of both compounds were studied in different solvents and as polymer-solvent blends. The photophysical properties of both boranes are evaluated and discussed in terms of their solvent-solute interactions using photoluminescence (PL) and NMR spectroscopies. The UV degradability of prepared thin films was studied by fluorimetric measurement. The effect of the surrounding atmosphere, dopant concentration and the molecular structure were assessed.

## 1. Introduction

Organic light emitting diodes (OLEDs) are an evolving technology suitable for a wide range of applications, such as high contrast displays in laptops, smartphones, TV, and smartwatches. The main advantage of OLEDs is their homogeneous emission over a large area and an internal charge to photon conversion efficiency that is now nearly 100%, a remarkable progress from the 25% efficiencies of the first OLEDs demonstrated in the year 1987 [1,2,3]. OLEDs are usually constructed from organic semiconductors deposited between carrier transporting layers with electrodes and require that charges be injected from the opposite electrodes into the emission layer in order to emit photons from the multilayered device. There is a contemporary research interest in the fabrication of individual layers from new materials, such as novel organic emitters embedded in conductive polymers, that combine to give good workability and to offer high stability, good external quantum efficiency, high brightness, and low cost [4,5,6]. The incorporation of heavy-metal atoms into organic molecules often facilitates phosphorescence and has led to improved efficiency. This approach is most successful in red and green OLEDs [7,8]. An approach to improve the lifetime of OLEDs is the utilization of nanocomposite active layers based on polymer matrices and semiconductive nanoparticle fillers. The charge carrier ratio is well balanced in such composites, which results in improved external quantum efficiency [9,10,11].

Despite the progress that has been made in this field, some challenges remain. For example, the broad and asymmetric emission spectrum of OLEDs is a serious general issue that can play a negative role in the color rendering index, and the relatively low stability of available blue emitters limits the long-term use of these devices [12]. In this context, the recent discovery that certain boronhydride clusters are capable of photostable and ultra-efficient blue emission [13] opens new possibilities in OLED device fabrication. These boronhydride clusters are novel inorganic materials with unique molecular architectures based on 3-dimensional polyhedra [14,15,16]. They possess a relatively sharp room temperature emission band between 400 and 460 nm (depending on the molecular structure and cluster substituents [17,18,19], and they have been shown to be air-stable as well as having good solubility in common solvents and some polymer matrices—all convenient properties for device fabrication. Thus far, the greatest attention has been paid to the cluster *anti*-B_18_H_22_ [13], which gives an emission of blue-purple light at 406 nm stable enough to act as the gain medium in the first borane-based laser [18]. More recently, a series of alkylated derivatives of *anti*-B_18_H_22_ have been reported that offer blue emission at around 425 nm with quantum yields of unity [19]. We are interested in investigating the feasibility of using the emission from these borane clusters as an active source for blue electroluminescence (EL) devices. Molecules and polymers with π-electron delocalized systems are standard material bases for organic or polymer electronics. A possibility to use polymers with σ-electron delocalization has been also demonstrated [20]. In contrast, due to their electron-deficient multicenter bonds, the development of borane clusters may launch a new class of electronic materials based on 3-dimensional electron delocalization and polyhedral molecular geometries. Indeed, these unique polyhedral geometries represent a bridge in the structural continuum from the condensed metallic assemblies to the open chains and rings of organic chemistry. As molecular structure has such an important bearing on functionality and molecular properties, then the boranes offer a novel resource for optical materials that we intend to delineate. One key aspect in this development is a thorough understanding of the photostability of luminescent borane materials in solid-state solution (i.e., in a polymer matrix). Although some work has already been done on their photoluminescence stability in solution [18,21], no such study has yet been made on their solid-state polymer-based materials, the absence of which is a bottleneck in the utilization of any borane-based active layer in OLEDs, where long-term stability of component materials is essential to device operation and market success [22,23,24,25].

**Figure 1:**
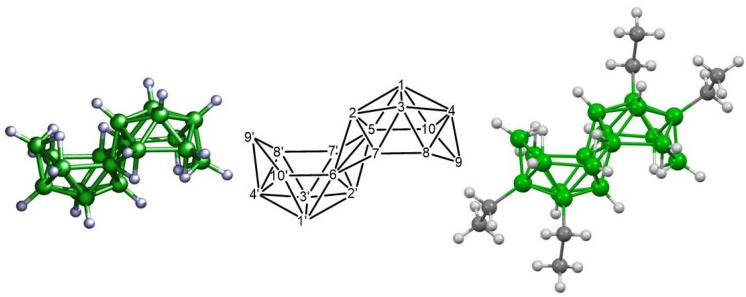
The molecular structure of 3,3′,4,4′-Et_4_-*anti*-B_18_H_18_ (compound **2**, right) [19]. In *anti*-B_18_H_22_ (compound **1**, left), the ethyl group positions are occupied by hydrogen atoms. The numbering system for the boron cluster is shown in the centre.

In this paper, we examine the quantum yield of luminescence (QY) and photostability of *anti*-B_18_H_22_ (compound **1**) and its tetraethylated derivative 3,3′,4,4′-Et_4_-*anti*-B_18_H_18_ (compound **2**) (see Figure 1) in solution (cyclohexane, toluene and chloroform) as well as in their borane/polymer composite blends. Polystyrene (PS) was chosen as the model polymer matrix since the preservation of the luminescent properties of borane compounds in polystyrene films bodes well for their potential use in optical devices [18,19]. An experimental study of the PS/borane blend films was designed to study their photodegradation in both air and in vacuum when irradiated with monochromatic light of a wavelength corresponding to the excitation maximum of the respective borane. The photodegradation of compounds **1** and **2** (and their composite polymer materials) was monitored by the decrease in their photoluminescence induced by various irradiation intensities/total energy doses. The results from this study provide valuable information to the key question: could boranes play an important role in novel OLED devices? We suggest that it may be possible to design suitable long-lasting borane-based OLEDs if the mechanism of degradation and photostability of these novel emitters is fully understood.

## 2. Materials and Methods

### 2.1. Preparation of Substrates

Quartz glass substrates were cleaned using a 4-step procedure in a series of solvents supported by sonication in an ultrasonic bath for at least 40 min. An aqueous solution of alkaline concentrate Hellmanex (2% of Hellmanex in demi-water) was used in the first step. Substrates were thoroughly rinsed in demi-water (0.07 μS/cm) afterwards and washed sequentially in acetone (p.a.) and isopropyl alcohol (p.a.). Substrates were then dried in a vacuum oven at 150 °C and stored until used. The surfaces were activated with ozone cleaner before use.

### 2.2. Sample Preparation and Characterization

Solutions of compounds **1** and **2** were prepared in cyclohexane (p.a.), chloroform (HPLC quality), and toluene (p.a.). All solvents were stored under a nitrogen atmosphere and dried over molecular sieves. A series of spin-coated thin films were prepared from a solution of Polystyrene Sigma Aldrich average Mw—350,000, Mn—170,000 P doped with molecules of **1** (Mn = 216.58 g mol^−1^) and **2** (Mn = 328.66 g mol^−1^), both of which were synthesized at the Institute of Inorganic Chemistry of the AS CR [19]. The polymer/borane blend solutions were cast onto quartz glass substrates by micropipette to spread an exact amount of material using spin coater Laurell WS-650-MZ-23NPP (Laurell Technologies Corporation, North Wales, USA) with a rotation speed of 1000 rpm. The thickness of the cast film depended on the solvent used. A thickness of ca 700 nm was observed for films cast from chloroform solution and ca 200 nm for films cast from toluene solution. These samples were used for both QY and degradability studies.

The solubility of polystyrene in cyclohexane is limited under the conditions employed. Therefore, Sigma Aldrich polystyrene, average Mw—35,000, was used. These solutions were used for the preparation of the thin films used for QY measurement only. The overall poor processability and quality of thin polystyrene films cast from cyclohexane solutions discounted them from being considered in the degradation study.

The films were dried at 50 °C in vacuum for two days. The drying temperature and time were verified as safe with regard to the thermal and migration stability of boranes in polymer films by online monitoring FTIR spectra.

The molar concentrations of both **1** and **2** were kept at 9 and 0.4 mmol/L in the solutions used for spin coating. The concentration of PS was kept 1.9 wt% in the solutions used for spin coating. Thus, an appropriate mass ratio between polymer and boranes was achieved. In the case of **1**, samples with polymer-borane mass ratios of 9:1 and 214:1 were prepared. For **2**, samples with polymer-borane mass ratios of 6:1 and 150:1, respectively, were prepared. In the case of cyclohexane, only the 0.4 mmol/L solutions with polymer-borane mass ratio 214:1 for **1** and 150:1 for **2** were used. These mass ratios result in the same PS monomeric unit to borane dopant molecule ratios of ca. 19:1 and ca. 460:1 for both borane compounds in thin films.

The whole process of sample preparation was performed in a nitrogen atmosphere in a glove box, and the prepared samples were kept in the dark before measurement.

Film thickness was measured by a mechanical profilometer Dektak XT-E (Bruker, Ettlingen, Germany) with a 1 nm resolution and by optical profilometry CONTOUR GT-K (Bruker, Ettlingen, Germany). Variable-angle spectroscopic ellipsometry UVISEL 2 (Horiba ABX SAS, Paris, France) was used for confirmation of film thickness.

The UV-VIS-NIR spectra were carried out using a double-beam spectrophotometer Lambda 1050 (Perkin Elmer, Louisville, KY, USA).

### 2.3. Quantum Yield Determination and Photostability Experiment

Photoluminescence (PL) spectra and PL intensity decreases were collected on a FLS920 fluorimeter (Edinburgh Instruments, Edinburgh, Scotland) in air, and in a vacuum (pressure 1Pa) ensured by the cryostat Optistat DN-V LN2 (Oxford Instruments, Oxford, UK), Oxford Instruments. An integrating sphere method was used for the determination of quantum yields of luminescence for both thin films and solutions. All measurements were carried out at room temperature.

To evaluate the dose of UV energy received over the surface of a sample, the intensity of monochromatic light was measured with a high-precision radiometer RM22 (Opsytec Dr. Gröbel, Ettlingen, Germany) equipped with the UV-A sensor. The size of the irradiated area was calculated from a digital image of graph paper with a visible ray trace. A UV fluorescent marker was used for the visualization of the light beam spot. The calculated powers of the radiating monochromatic light with a wavelength of 340 nm depending on the slit size are shown in Table 1.

## 3. Results and Discussion

### 3.1. Photoluminescent Properties in Solution

**Figure 2:**
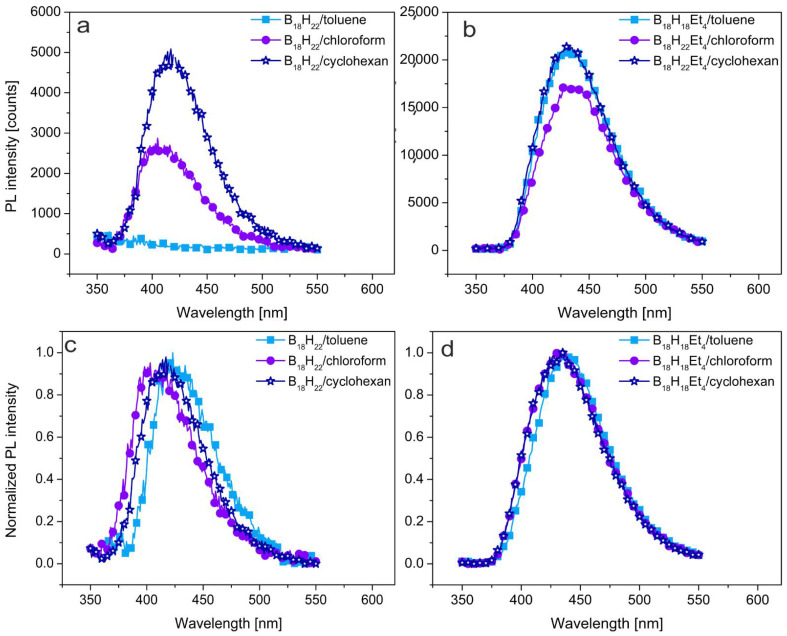
Photoluminescence emission spectra of (**a**) *anti*-B_18_H_22_ and (**b**) 3,3′,4,4′-Et_4_-*anti*-B_18_H_18_ in solutions, (**c**,**d**) are their normalized form. Excitation wavelength λ_exc_ = 340 nm for all samples.

The quantum yield of luminescence (QY, defined as a ratio of the number of emitted photons to the number of photons absorbed by the sample) of **1** in cyclohexane was reported to have a value close to unity [18]. Our independent measurements, which repeatedly gave a value of 0.96, confirm this property. However, we also found significantly different QYs for **1** in other solvents (summarized in Table 2). Thus, measurements of solutions of **1** in chloroform show a drop in QY to 0.88, and toluene solutions record a collapse in QY to 0.05 and a redshift of the emission maximum as illustrated in Figure 2. In an interesting contrast, compound **2** exhibits a high QY in all the solvents used, with toluene solutions giving a QY of 0.95, and a roughly constant emission maximum (Figure 2). This suggests that although the borane cluster photoactive core of compounds **1** and **2** are very similar, the presence of ethyl substituents in compound **2** significantly alter solvate-solvent interactions.

In addition to the data in Table 2, measured values for the QYs of both **1** and **2** mixed with polystyrene in various solvents are summarized in Table 3. This data provides information on the relevance of the borane luminophore-polystyrene interaction on the QY whilst still in solution.

Clearly, there is a fundamental influence of the solvent on the PL of borane compound **1** that is not nearly as pronounced for **2**. The highest values of QY for **1** and **2** were, in both cases, obtained when measured in cyclohexane solution. This suggests that the saturated aliphatic hydrocarbon solvent has no adverse effect on the PL of either compound. Both compounds offer 10–15% lower QY in chloroform, which is a polar solvent conceivably capable of inducing temporary dipole moments in compounds **1** and **2** that could affect their molecular orbital systems and, hence, the PL of the luminophores. However, it is when dissolved in toluene that we see the most remarkable difference. Whereas the QY for compound **2** hardly changes in toluene, in the case of **1,** the QY almost completely collapses, reducing by over 90%. Clearly, the four ethyl substituents on the octadecaborane cluster of **2** prevent an interaction with toluene that is able to profoundly manifest itself with **1**.

Nuclear Magnetic Resonance (NMR) spectroscopy is a good tool to investigate potential solute-solvent interactions. Using this technique on various solutions of B_10_H_14_, Gibb and Kennedy proposed, from a measurement of relaxation times of the boron and proton resonances, a significant non-classical interaction between the decaborane cluster and the π-electron cloud of the aromatic solvent [26]. Later Fontaine and Kennedy et al. [27] noted a large aromatic-solvent induced shielding for the H(8,9) bridging proton in *anti*-B_18_H_22_ and again postulated a weak interaction or molecular complex to explain the observation [27].

**Figure 3:**
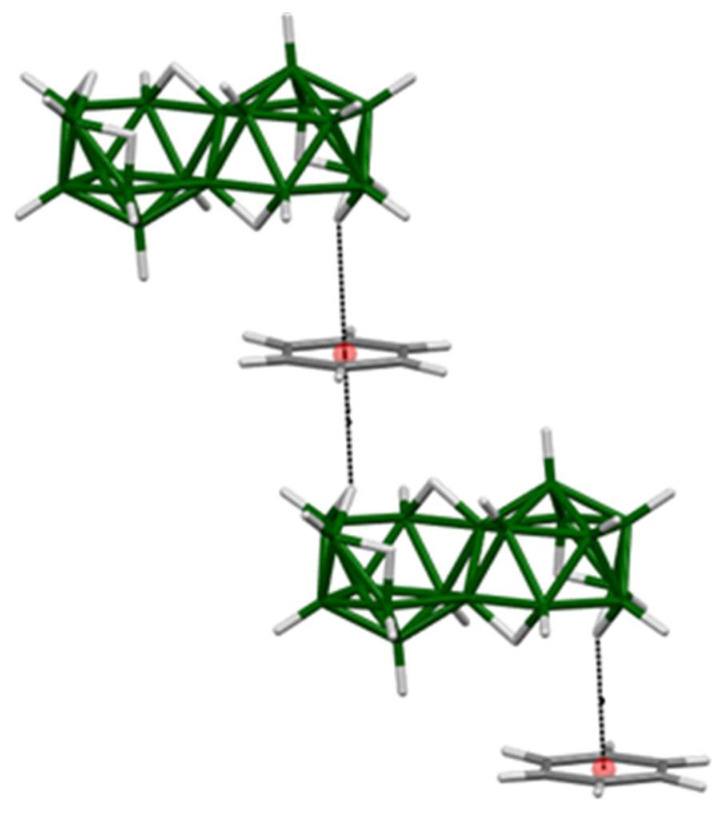
The crystal structure of the *anti*-B_18_H_22_—benzene solvate [28] with *anti*-B_18_H_22_···benzene stacks arising from interaction between the H(8,9) bridging hydrogen atom and the π-cloud of the benzene ring.

Bridging hydrogen atoms in neutral borane molecules are known to be acidic in character and, therefore, adopt a partial positive charge, which is in contrast to the hydridic nature of the terminally bonded cluster hydrogen atoms. Therefore, such a bridging hydrogen atom···π-electron cloud interaction is a reasonable hypothesis. Hamilton and Shore later confirmed this hypothesis [28] by providing x-ray crystallographic evidence of *anti*-B_18_H_22_···benzene stacks in the crystal structure of *anti*-B_18_H_22_·C_6_H_6_ that clearly show short-contact interactions between H(8,9) and H(8′,9′) bridging hydrogen atoms in *anti*-B_18_H_22_ with the π-electron clouds in molecules of benzene as shown in Figure 3.

**Figure 4:**
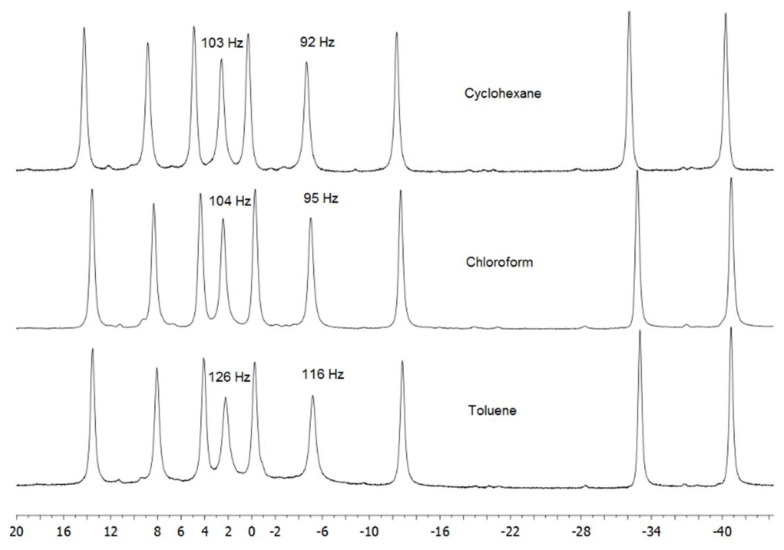
The ^11^B-{^1^H} NMR spectra of *anti*-B_18_H_22_ in three solvents. The two resonances assigned to the B8 and B9 boron atoms exhibit the largest broadening in the aromatic solvent.

As NMR is sensitive to changes to molecular orbital (MO) systems in a way not dissimilar to how the photophysics of luminescent molecules is determined by relative HOMO/LUMO energies, it is our conjecture that the explanation for the change in NMR properties of **1**, detailed above, is indeed the same rationale for the dramatic decrease in QY that we observe for its toluene solution, i.e., that the formation of H(8,9) atom···π-electron interactions quenches the fluorescence of **1**. Interestingly, the ^11^B NMR of compound **1** in toluene solvent shows significant line-broadening in the majority of resonances, in particular for atoms B(8) and B(9), across which spans the bridging hydrogen atom H(8,9) (Figure 4). Such line broadening, caused by faster relaxation times, is indicative of less efficient molecular tumbling in solution, which would be an anticipated manifestation of *anti*-B_18_H_22_···toluene aggregations.

**Figure 5:**
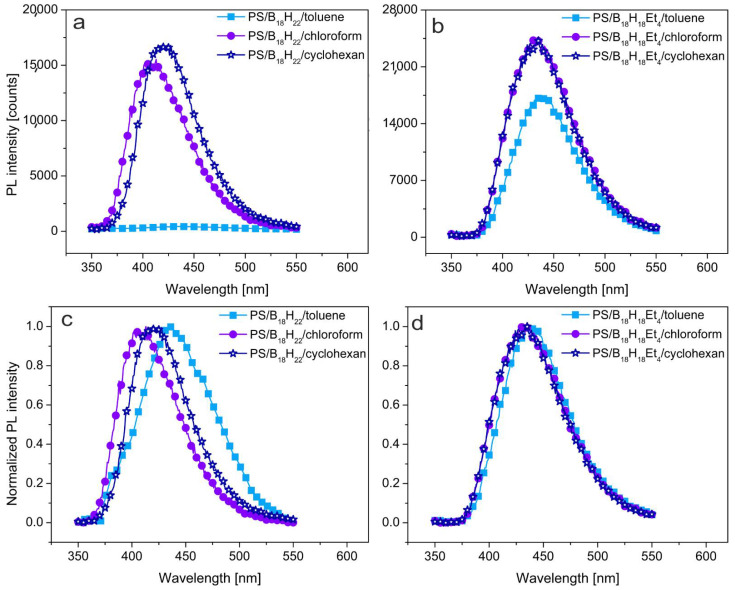
Photoluminescence emission spectra of (**a**) *anti*-B_18_H_22_ and (**b**) 3,3′,4,4′-Et_4_-*anti*-B_18_H_18_ in solutions with polystyrene, (**c**,**d**) are their normalized form. Excitation wavelength λ_exc_ = 340 nm for all samples.

The ethyl substituents on compound **2** have been shown [19,29] to be electron-withdrawing from the borane cluster, resulting in a reduced acidity for the cluster bridging hydrogen atoms. This effect may be sufficient to inhibit solvate-solvent interaction, and consequently, the QY of luminescence remains almost the same in toluene as in cyclohexane solution. Interestingly, in the case of mixtures of compound **2** with polystyrene (PS), the presence of the polymer eliminates the mitigating effect of chloroform solvent on the QY of the system. Such subtleties, along with information on shifting emission wavelength maxima, are graphically projected in Figure 5, where the PL emission spectra of **1** and **2** as PS blends are plotted. In the case of **1**, PL intensity is shown to drastically decrease in toluene. Furthermore, in the normalized PL spectra, a measurable redshift of emission maximum by up to 30 nm is evident, that seems to reflect the trend in the proton affinity (PA) of solvents (PA, in kJ·mol^−1^ of used solvents are: toluene—784, cyclohexane—687, chloroform—664 [30]). Such a redshift for **1** is presumably also linked to the extended MO systems consequent of the B-H(8,9)-B···π-stacking described above. It should be noted that a full proton transfer from cluster to solvent has not been observed under any circumstance, as followed by NMR spectroscopy, and it is highly unlikely to occur in relatively non-polar solvents. In contrast, compound **2** maintains a QY close to unity in all the solvents used, and the decrease in PL intensity is small with no observed redshift of the emission maxima. Thus, the ethylation of the photoactive 18-vertex borane cluster shows a potentially promising avenue to the improvement of photoluminescence stability, even in an environment with high proton affinity. This may be a crucial finding, since all electronic materials derived from π-delocalized electron bonding systems often have relatively high PAs, which would be detrimental for the use of the unprotected borane **1** as a functional molecular additive to these matrices.

### 3.2. Photoluminescent Properties in Polystyrene Thin Films

Values of luminescence QY for boranes **1** and **2** incorporated as molecular additives in thin polystyrene films that were dried and free of used solvents were also obtained and are summarized in Table 4.

It is important to note that the monomer analogue of the polystyrene matrix used is ethylbenzene and this has a proton affinity (PA) of 788 kJ·mol^−1^. Another monomer analogue is isopropyl benzene with PA 791.6 kJ·mol^−1^ [30]. Thus, a similar PA value may be assumed for the polystyrene monomer unit. Moreover, these PA values are slightly higher than for toluene. Such a relatively high PA value could support the creation of a π-stacking complexes of boranes **1** and **2** in PS matrices similar to those described above, and therefore a decrease in their QY might be expected. This was, indeed, our eventual observation for thin polystyrene films cast from cyclohexane and toluene solutions, the latter being the worst case. The decrease in QY was more pronounced in the case of **1**. If the thin films were prepared from solutions in chloroform then decrease in QY was smaller, only about 30%. Based on this, we suggest that, in the case of chloroform, the charge redistributions caused by interactions of borane hydrogen atoms with phenyl side groups of polystyrene is much smaller. Although it seems to be highly probable that chloroform has a positive effect on QY of the boranes in solution due to the smaller proton affinity of chloroform [31,32,33,34], the solid-state effect is manifested after removal of the solvent. Surprisingly, chloroform is a better solvent for polystyrene than toluene and cyclohexane. Their Hansen parameter for nonpolar interactions are nearly the same (*δ_d_* of chloroform, chlorobenzene, toluene: 17.8, 16.8, 18.0), the dipolar interactions (*δ_p_*-chloroform, chlorobenzene, toluene: 3.1, 0.0, 1.4) and the hydrogen-bonding interactions (*δ_h_*-chloroform, chlorobenzene, toluene: 5.7, 0.2, 2.0) are about two times higher for chloroform than those for the other two solvents [35]. It can be hypothesized that there is competition between chloroform-polystyrene and borane-polystyrene interactions with the dominance of the former affecting the development of intermolecular interactions in the borane-polymer systems during thin film drying. The stronger affinity of chloroform to polystyrene may block the development of the undesirable proton transfer interaction until the very last stage of the film formation when the polymer conformation is locked in the almost dry film, and the geometry sensitive B-H···π-stacking interaction cannot develop after the chloroform has completely evaporated. It seems that a proper selection of the solvent system may avoid the weakness of the borane **1**, at least to a certain extent.

We also studied the photoluminescence stability of these materials when incorporated in polymer thin films, as we intend to use borane molecules in thin polymer films as the dopant active emitter in OLEDs, and photostability is one of the critical issues for such an application. If these materials were to have comparably high photostability in thin films as has been already reported for *anti*-B_18_H_22_ in solution under laser irradiation, which are superior to many of the commercially available state-of-the-art blue laser dyes [18], it will be possible to introduce a whole new class of emitters. Nevertheless, it seems to be not as straightforward as we had anticipated, and an intriguing yet subtle, rather than destructive, photodegradation chemistry may obstruct the application [25]. To investigate the role of borane-borane molecule interactions, two levels of dopant concentration in the solid polymer film were examined. The more highly concentrated material represents the case of dopant molecules in close proximity, while the less concentrated case represents luminophore molecules isolated in the polymer matrix. This assumption was verified by the PL emission spectra recorded for thin films as discussed below. As a useful tool for the photostability characterization of materials in the form of thin films, the approach using fluorescence spectroscopy and the monitoring of photoluminescence decrease may be successfully applied [36,37]. The study was performed in two different environments. One set of experiments was carried out in a vacuum, in order to simulate encapsulation or other protection of the device, and a second set in air to represent exposure to the ambient atmosphere. To investigate the degradation process of compounds **1** and **2**, the PL decrease was continuously monitored in 1 s intervals while the sample surfaces were irradiated by energies of 0.20, 0.46, and 0.72 mW·cm^−2^, respectively.

**Figure 6:**
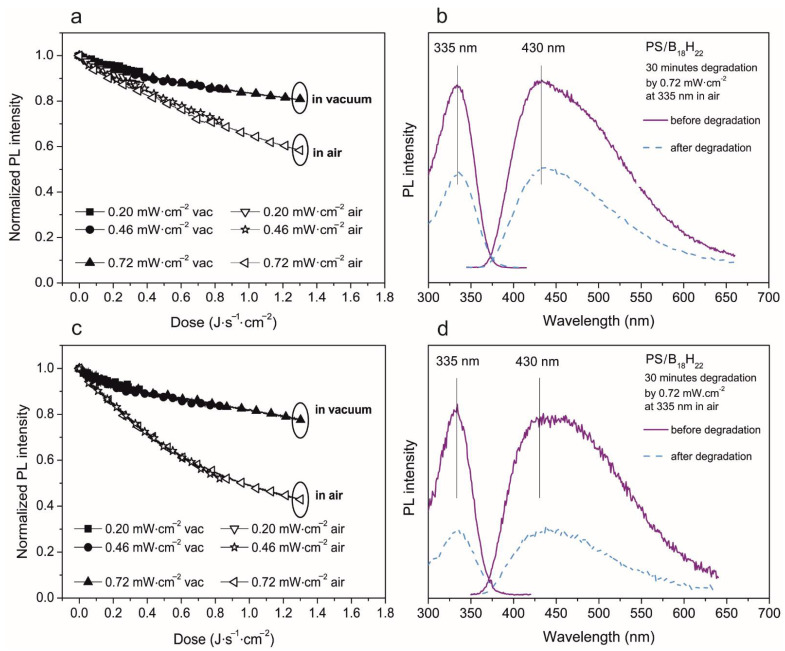
Photodegradation study of 750 nm (**a**,**b**) and 200 nm (**c**,**d**) thick film of PS/*anti*-B_18_H_22_ in ratio 9:1. Left (**a**,**c**)—evolution of normalized PL intensity of emission maxima in both vacuum and air environment depending on the energy dose. The excitation and emission spectra measured before and after 30 min of degradation—right (**b**,**d**).

In Figure 6, left, the PL intensity decrease curves of the PS/*anti*-B_18_H_22_ blend in a 9:1 ratio are depicted. Part (a) represents a thick film of about 750 nm and (b) represents a thinner film with a thickness of about 200 nm. It is clearly seen in the case of thicker films, that the total PL intensity decrease relative to the initial value for the highest degradation dose is 20% in a vacuum and 40% in air. Additionally, in the case of the thin film, the PL intensity decrease keeps the value 20% in a vacuum, but it is about 60% in air. With regard to emission and excitation spectra, no change was observed, with only an overall decrease in PL intensity. This fact is in direct contrast to our expectation based on earlier work [18], where superior photo-stability was observed, even comparable with an Exalite 404 laser dye. Our particular observation illustrated in Figure 6 indicated borane degradation in PS matrix although it was pumped by a much weaker and non-coherent source (Xe lamp) in a vacuum. The degradation was accelerated when humidity and air were present. On the other hand, the solvent used for thin film preparation or the film thickness does not seem to play a significant role in the PL intensity decrease.

**Figure 7:**
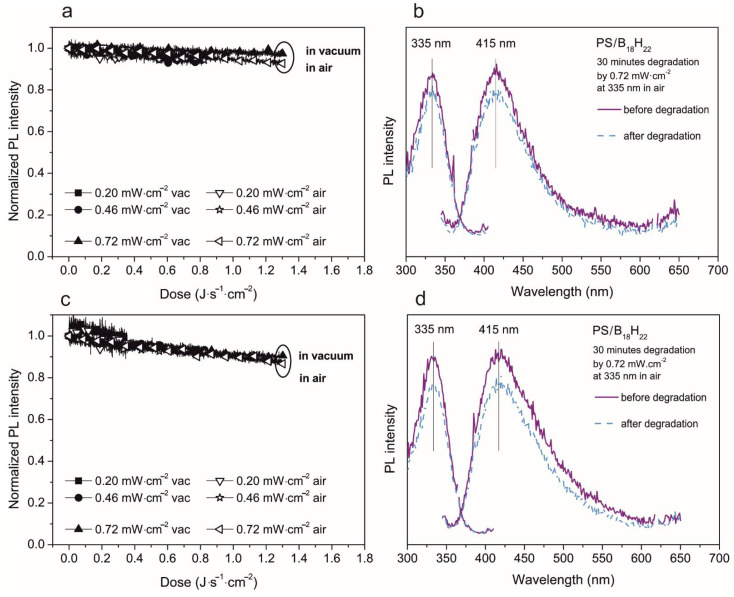
Photodegradation study of 750 nm (**a**,**b**) and 200 nm (**c**,**d**) thick film of PS/*anti*-B_18_H_22_ in ratio 214:1. Left (**a**,**c**)—evolution of normalized PL intensity of emission maxima in both vacuum and air environment depending on the energy dose. The excitation and emission spectra measured before and after 30 min of degradation—right (**b**,**d**).

This situation changes, however, when the weight ratio of PS to borane is different. The PL intensity decrease is much smaller if the PS/borane weight ratio was 214:1. A summary of our measurements of these films are presented in Figure 7. For thicker films, the PL intensity decrease for the highest degradation dose is only 5% in a vacuum and 10% in air, relative to the initial value. For thin films, the PL intensity decrease is 10% in both the vacuum and in air. Additionally, here, the solvent or film thickness used seems not to be important in the photodegradation.

Additionally, in the case of the different weight ratio between polystyrene and borane, no difference in emission and excitation spectra was observed before or after degradation, although, the emission maximum and shape of emission spectra do differ between films with different weight ratios. For films with the lower loading of borane, the emission maximum is at the same wavelength as when the borane is in cyclohexane solution. In more densely loaded films, the emission maximum is redshifted, and the spectrum is broader. The redshift and broadening of the emission peak could be due to conglomeration via H-bridge interactions giving rise to an extended stacked structure [28]. Another possibility for high concentration blends, where individual borane clusters are in close proximity to each other, is self-absorption of irradiated energy. This borane-borane intermolecular photophysical interaction, involving excited state absorption of emitted light, might be responsible for observed photoluminescence decrease, at least partially. Some indications of such behavior were observed for laser pumped PL experiments [25].

**Figure 8:**
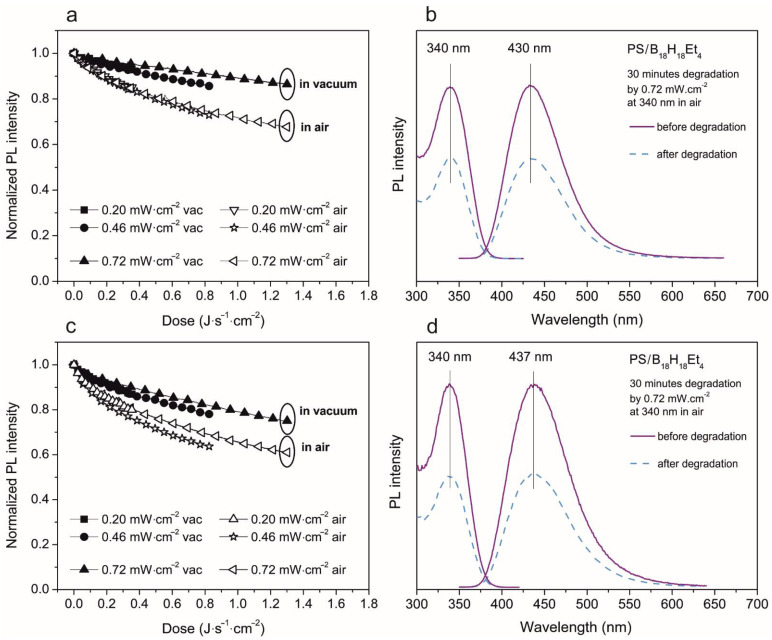
Photodegradation study of 750 nm (**a**,**b**) and 200 nm (**c**,**d**) thick film of PS/3,3′,4,4′-Et_4_-*anti*-B_18_H_18_ in weight ratio 6:1. Left (**a**,**c**)—evolution of normalized PL intensity of emission maxima in both vacuum and air environment depending on the energy dose. The excitation and emission spectra measured before and after 30 min of degradation—right (**b**,**d**).

**Figure 9:**
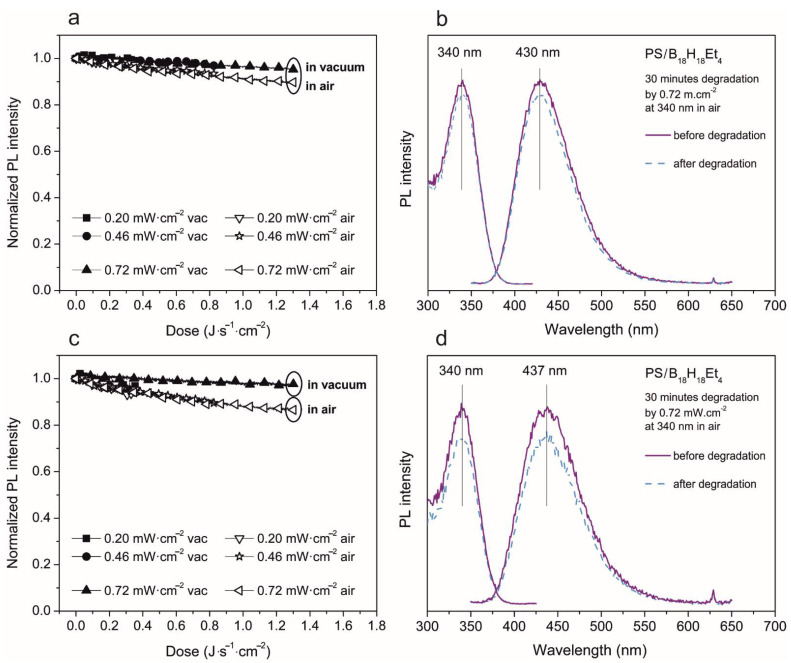
Photodegradation study of 750 nm (**a**,**b**) and 200 nm (**c**,**d**) thick film of PS/3,3′,4,4′-Et_4_-*anti*-B_18_H_18_ in ratio 150:1. Left (**a**,**c**)—evolution of normalized PL intensity of emission maxima in both vacuum and air environment depending on the energy dose. The excitation and emission spectra measured before and after 30 min of degradation—right (**b**,**d**).

If we compare the PL intensity decrease decay of **1** and **2**, the ethylated borane shows better stability. Its PL intensity decrease is lower in all the studied cases: for thin and thick films with a high amount of borane in the PS/borane blend (results are depicted in Figure 8), and also for thin and thick films with a lower amount of borane in PS/borane blend (results presented in Figure 9). Thicker films, prepared from a PS/ethylated borane blend with a ratio 6:1, show PL intensity decrease when using the highest degradation energy of 10% in a vacuum and30% in air. In the case of thinner films, PL intensity degradation reaches 25% in a vacuum and 40% in air. The general shapes of PL spectra and their maxima are not changed. In films with lower loading of **2** in the blend, the PL intensity decrease is very small, only 5% in a vacuum (valid for both thick and thin films) and only 10% in air in the case of thick films and 15% for thin films. Additionally, in this case, the solvent and film thickness does not significantly affect the PL degradation process.

The obvious trend is that the ethylation of *anti*-B_18_H_22_ provides a luminescent molecule with several advantages for use in thin-layer fabrication. To further stabilize borane molecules, there exist different strategies. One can be the inclusion of borane clusters in the cavity of a protective host, e.g., cyclodextrins [38]. Another is the introduction of alkyl substituents on the borane cage skeleton. Extensive methylation, for example, increases the usefulness of these compounds significantly, as published recently [29], and our work with ethylated compounds confirmed this approach viable. Moreover, besides stabilization, the choice of the substituents offers the prospect of the engineering of the PL emission wavelength and allows the hope to synthesize a full-color palette of emitters [19,39].

**Figure 10:**
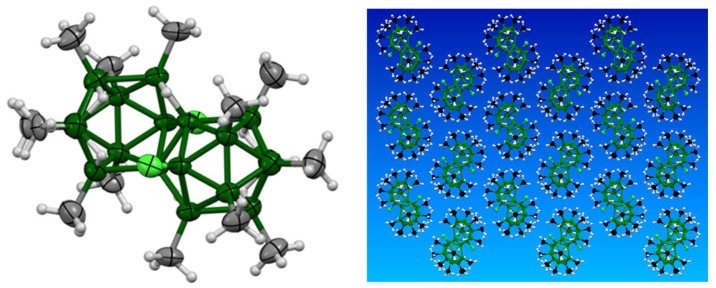
The molecular structure of *anti*-B_18_H_7_Cl_2_Me_13_ containing 13 peripheral methyl groups and two chlorine atoms [29]. The right view shows the crystal packing for the molecule and emphasizes the potentially protective sheath of methyl groups around the boron core (green atoms).

Here, it may be noted that increasing the number of alkyl substituents on the borane cage may improve its stability in coordinating solvents, and thence in polymer matrices. Small amounts of compounds containing up to 13 methyl groups, out of a potential maximum of 16, have been identified, as in, for example, *anti*-B_18_H_7_Cl_2_Me_13_ [29] (Figure 10). These highly alkylated clusters show a greatly reduced acidity compared to *anti*-B_18_H_22_ **1**, which easily loses one proton in solution with the mild base Proton Sponge [40] and a second proton with the stronger base sodium hydride, NaH. However, we have found [unpublished observations] that, for example, *anti*-B_18_H_8_Cl_2_Me_12_ requires NaH to remove the first proton and the second proton cannot now be removed by NaH. Thus, these materials may have a considerably reduced interaction with the polymer matrix, and it would be useful to investigate the use of these compounds if higher yield synthetic routes can be developed.

## 4. Conclusions

We have investigated the photostability of *anti*-B_18_H_22_ and *anti*-B_18_H_18_Et_4_ boranes in solutions and incorporated as dopants in polymer matrices at various concentrations. The study was performed by a determination of the quantum yield of luminescence (QY) and by direct on-line measurement of the PL decrease, simultaneously using the beam for excitation and as the degradation stress. The elimination of air and humidity can suppress the photodegradation of presented boranes to a reasonable extent. However, the high proton affinity of the solvents, which is an intrinsic property, limits the QY of *anti*-B_18_H_22_ due to solvent-solute interactions. Moreover, the bridging hydrogens are prone to the formation of π-stacking interactions not only in liquid solutions but also in solid solutions, which is responsible for the poor PL of thin PS films prepared from toluene solutions. These interactions must therefore be either inactivated or the cluster properties modified by the introduction of boron cage substituents. The former approach was proven by a selection of an appropriate solvent to avoid the formation of such hydrogen bond configurations in drying polymer films. The latter approach was proven as superior under all tested circumstances in the case of 3,3′,4,4′-Et_4_-*anti*-B_18_H_18_. Without proper stabilization, it would not be possible to combine borane dopants with materials based on delocalized π-bonding systems.

However, the borane-polymer blends (as exemplified on polystyrene) currently available still suffer from photodegradation to a certain extent, even in an inert environment (vacuum)—including the blend with 3,3′,4,4′-Et_4_-*anti*-B_18_H_18_. It was shown that concentration of the borane in the polymer matrix can be a critical issue as well. The borane–borane interaction, possibly in an excited state, might also be partly responsible for observed photoluminescence decrease. On the other hand, the sample solvent processing history does not seem to influence the PL degradation rate, although it has dramatic effect on the QY. Nevertheless, our present results together with the photostability studies performed in solutions that are available in literature, give reason to hope for further improvement of the stability of the borane molecules in polymer blends. Solving this problem will unlock the application of the whole new family of borane based alternative molecular components in the fabrication of light-emitting devices.

## Figures and Tables

**Table 1:** Recalculated values of irradiation power used for degradation experiments.

Slit Size [μm]	UV Irradiance [mW·cm^−2^]
1	0.20
2	0.46
3	0.72

**Table 2:** QY of borane compounds in different solvents.

Solvent	QY		Emission Maxima (nm)	
	*anti*-B_18_H_22_	3,3′,4,4′-Et_4_-*anti*-B_18_H_18_	*anti*-B_18_H_22_	3,3′,4,4′-Et_4_-*anti*-B_18_H_18_
Cyclohexane	0.96 ± 0.03	0.99 ± 0.01	417	434
Chloroform	0.88 ± 0.03	0.85 ± 0.02	407	434
Toluene	0.05 ± 0.01	0.95 ± 0.02	425	439

**Table 3:** QY of borane compounds in different solvents mixed with polystyrene solutions.

Solvent	QY		Emission Maxima (nm)	
	*anti*-B_18_H_22_	3,3′,4,4′-Et_4_-*anti*-B_18_H_18_	*anti*-B_18_H_22_	3,3′,4,4′-Et_4_-*anti*-B_18_H_18_
Cyclohexane	0.82 ± 0.05	0.99 ± 0.01	420	434
Chloroform	0.73 ± 0.02	0.99 ± 0.01	409	434
Toluene	0.03 ± 0.02	0.95 ± 0.02	437	439

**Table 4:** QY of borane compounds in a polystyrene matrix in the form of thin film cast from various solvents.

Solvent Used for Thin Film Preparation	QY		Emission Maxima (nm)	
	*anti*-B_18_H_22_	3,3′,4,4′-Et_4_-*anti*-B_18_H_18_	*anti*-B_18_H_22_	3,3′,4,4′-Et_4_-*anti*-B_18_H_18_
Cyclohexane	0.13 ± 0.03	0.22 ± 0.04	415	432
Chloroform	0.67 ± 0.02	0.69 ± 0.06	405	436
Toluene	0.07 ± 0.04	0.29 ± 0.05	432	433

## Data Availability

Data and methods used are presented in sufficient detail in the paper.

## References

[1] Goushi K., Yoshida K., Sato K., Adachi C. (2012). Organic light-emitting diodes employing efficient reverse intersystem crossing for triplet-to-singlet state conversion. Nat. Photonics.

[2] Lee J.-H., Chen C.-H., Lee P.-H., Lin H.-Y., Leung M.-K., Chiu T.-L., Lin C.-F. (2019). Blue organic light-emitting diodes: Current status, challenges, and future outlook. J. Mater. Chem. C.

[3] Li W., Pan Y., Xiao R., Peng Q., Zhang S., Ma D., Li F., Shen F., Wang Y., Yang B. (2014). Employing ∼100% Excitons in OLEDs by Utilizing a Fluorescent Molecule with Hybridized Local and Charge-Transfer Excited State. Adv. Funct. Mater..

[4] Yang Z., Gao M., Wu W., Yang X., Sun X.W., Zhang J., Wang H.-C., Liu R.-S., Han C.-Y., Yang H. (2019). Recent advances in quantum dot-based light-emitting devices: Challenges and possible solutions. Mater. Today.

[5] Giovanella U., Pasini M., Botta C. (2016). Organic Light-Emitting Diodes (OLEDs): Working Principles and Device Technology.

[6] Anikeeva P.O., Madigan C.F., Halpert J.E., Bawendi M., Bulović V. (2008). Electronic and excitonic processes in light-emitting devices based on organic materials and colloidal quantum dots. Phys. Rev. B.

[7] Yang X., Zhou G., Wong W.-Y. (2016). ChemInform Abstract: Functionalization of Phosphorescent Emitters and Their Host Materials by Main-Group Elements for Phosphorescent Organic Light-Emitting Devices. Chem. Soc. Rev..

[8] Ho C.-L., Li H., Wong W.-Y. (2014). Red to near-infrared organometallic phosphorescent dyes for OLED applications. J. Organomet. Chem..

[9] Jamatia T., Škoda D., Urbanek P., Sevcik J., Maslik J., Munster L., Kalina L., Kuřitka I. (2019). Microwave-assisted synthesis of FexZn1−xO nanoparticles for use in MEH-PPV nanocomposites and their application in polymer light-emitting diodes. J. Mater. Sci. Mater. Electron..

[10] Škoda D., Urbanek P., Sevcik J., Munster L., Nádaždy V., Cullen D.A., Bazant P., Antos J., Kuritka I. (2018). Colloidal cobalt-doped ZnO nanoparticles by microwave-assisted synthesis and their utilization in thin composite layers with MEH-PPV as an electroluminescent material for polymer light emitting diodes. Org. Electron..

[11] Škoda D., Urbánek P., Sevcik J., Munster L., Antos J., Kuřitka I. (2018). Microwave-assisted synthesis of colloidal ZnO nanocrystals and their utilization in improving polymer light emitting diodes efficiency. Mater. Sci. Eng. B.

[12] Lee H., Maeng M.-J., Hong J.-A., Najnin R., Moon J., Cho H., Lee J., Yu B.-G., Park Y., Cho N.S. (2017). Highly efficient green, blue, and white phosphorescent inverted organic light-emitting diodes by improving charge injection and balance. J. Mater. Chem. C.

[13] Londesborough M.G.S., Hnyk D., Bould J., Serrano-Andrés L., Sauri V., Oliva-Enrich J.M., Kubát P., Polívka T., Lang K. (2012). Distinct Photophysics of the Isomers of B_18_H_22_ Explained. Inorg. Chem..

[14] Cooke P.A., O’Dowd C., Londesborough M.G.S., Holub J., Štíbr B., Thornton-Pett M., Clegg W., Teat S.J., Kennedy J.D. (2000). B-frame supported bimetallics. “Composite cluster” compounds and the structures of [2,7-(η^5^-C_5_Me_5_)_2_-*nido*-2,7,8,6-Ir_2_CSB_6_H_8_] and its 9-chloro derivative. Synchrotron and conventional X-ray studies. J. Organomet. Chem..

[15] Saurí V., Oliva J.M., Hnyk D., Bould J., Braborec J., Merchán M., Kubát P., Císařová I., Lang K., Londesborough M.G.S. (2013). Tuning the Photophysical Properties of *anti*-B_18_H_22_: Efficient Intersystem Crossing between Excited Singlet and Triplet States in New 4,4′-(HS)_2_-*anti*-B_18_H_20_. Inorg. Chem..

[16] King R.B. (2001). Three-dimensional aromaticity in polyhedral boranes and related molecules. Chem. Rev..

[17] Londesborough M.G.S., Dolanský J., Cerdán L., Lang K., Jelínek T., Oliva J.M., Hnyk D., Roca-Sanjuán D., Francés-Monerris A., Martinčík J. (2017). Thermochromic Fluorescence from B_18_H_20_(NC_5_H_5_)_2_: An Inorganic–Organic Composite Luminescent Compound with an Unusual Molecular Geometry. Adv. Opt. Mater..

[18] Cerdán L., Braborec J., Garcia-Moreno I., Costela A., Londesborough M.G.S. (2015). A borane laser. Nat. Commun..

[19] Bould J., Lang K., Kirakci K., Cerdán L., Roca-Sanjuán D., Francés-Monerris A., Clegg W., Waddell P.G., Fuciman M., Polivka T. (2020). A Series of Ultra-Efficient Blue Borane Fluorophores. Inorg. Chem..

[20] Miller R.D., Michl J. (1989). Polysilane High Polymers. Chem. Rev..

[21] Tan C.H., Zhang B.K., Chen J., Zhang L.N., Huang X.G. (2019). Study of Hydrolysis Kinetic of New Laser Material [*anti*-B_18_H_22_]. Russ. J. Inorg. Chem..

[22] Lin N., Qiao J., Duan L., Wang L., Qiu Y. (2014). Molecular Understanding of the Chemical Stability of Organic Materials for OLEDs: A Comparative Study on Sulfonyl, Phosphine-Oxide, and Carbonyl-Containing Host Materials. J. Phys. Chem. C.

[23] Aziz H., Popovic Z.D. (2004). Degradation Phenomena in Small-Molecule Organic Light-Emitting Devices. Chem. Mater..

[24] So F., Kondakov D. (2010). Degradation mechanisms in small-molecule and polymer organic light-emitting diodes. Adv. Mater..

[25] Cerdán L., Francés-Monerris A., Roca-Sanjuán D., Bould J., Dolanský J., Fuciman M., Londesborough M.G.S. (2020). Unveiling the role of upper excited electronic states in the photochemistry and laser performance of: *Anti*-B_18_H_22_. J. Mater. Chem. C.

[26] Gibb T.C., Kennedy J.D. (1982). Proton and boron-11 nuclear spin relaxation and the molecular tumbling of nido-decaborane in perdeuterotoluene solution. An interesting transition in solute–solvent interaction behaviour. J. Chem. Soc..

[27] Fontaine X.L.R., Greenwood N.N., Kennedy J.D., MacKinnon P. (1988). Boron-11 and proton nuclear magnetic resonance study of *anti*-B_18_H_22_ and its anions, *anti*-[B_18_H_21_]^−^ and *anti*-[B_18_H_20_]^2−^. The crystal and molecular structure of [NMe_4_]_2_[*anti*-B_18_H_20_]. J. Chem. Soc. Dalton Trans..

[28] Hamilton E.J.M., Kultyshev R.G., Du B., Meyers E.A., Liu S., Hadad C.M., Shore S.G. (2006). A stacking interaction between a bridging hydrogen atom and aromatic π density in the n-B_18_H_22_-benzene system. Chem.-A Eur. J..

[29] Londesborough M.G.S., Lang K., Clegg W., Waddell P.G., Bould J. (2020). Swollen Polyhedral Volume of the *anti*-B_18_H_22_ Cluster via Extensive Methylation: *Anti*-B_18_H_8_Cl_2_Me_12_. Inorg. Chem..

[30] Hunter E.P.L., Lias S.G. (1998). Evaluated Gas Phase Basicities and Proton Affinities of Molecules: An Update. J. Phys. Chem. Ref. Data.

[31] Henderson D.J., Rettner C.T. (2003). Physical Chemistry. Encyclopedia of Physical Science and Technology.

[32] Barthel J., Neueder R. (2003). Chemical Thermodynamics. Encyclopedia of Physical Science and Technology.

[33] Karelson M., Shibuya T.I., Wielgus M., Bartkowiak W. (2014). Electronic and electrical effects of solvents. Handbook of Solvents.

[34] Safi Z.S., Omar S. (2014). Proton affinity and molecular basicity of m-and p-substituted benzamides in gas phase and in solution: A theoretical study. Chem. Phys. Lett..

[35] Hansen C.M. (2007). Hansen Solubility Parameters Second edition: A User’s Handbook.

[36] Kuřitka I., Schauer F., Sáha P., Zemek J., Jiricek P., Nešpůrek S. (2006). UV degradability of polysilanes for nanoresists examined by electron spectroscopies and photoluminescence. Czechoslov. J. Phys..

[37] Urbánek P., Kuřitka I. (2015). Thickness dependent structural ordering, degradation and metastability in polysilane thin films: A photoluminescence study on representative σ-conjugated polymers. J. Lumin..

[38] Tan C., Chen J., Zhang L., Zhang B., Huang X., Meng H. (2019). The preparation and characterization of n-B_18_H_22_-beta cyclodextrin inclusion complex. IOP Conference Series: Earth and Environmental Science.

[39] Londesborough M.G.S., Dolanský J., Jelínek T., Kennedy J.D., Císařová I., Kennedy R.D., Roca-Sanjuán D., Francés-Monerris A., Lang K., Clegg W. (2018). Substitution of the laser borane: *Anti*-B_18_H_22_ with pyridine: A structural and photophysical study of some unusually structured macropolyhedral boron hydrides. Dalton Trans..

[40] Alder R.W., Bryce M.R., Goode N.C., Miller N., Owen J. (1981). Preparation of a range of NNN′N′-tetrasubstituted-1,8-diaminonaphthalenes. J. Chem. Soc. Perkin Trans. 1.

